# Supplementary material for: Global gradients in intertidal species richness and functional groups
Source: eLife. 2021 Mar 19;10:e64541. doi: 10.7554/eLife.64541 (PMC8032391; doi:10.7554/eLife.64541)
Supplement: Supplementary file 1. [file elife-64541-supp1.docx]

**Supplementary File 1**: Number of sites in 5° latitudinal bands between -75° to 85° of latitude

| Latitudinal band | Number of sites | |
| --- | --- | --- |
| -75–71 | 1 |  |
| -70–66 | 7 |  |
| -65–61 | 10 |  |
| -60–56 | 2 |  |
| -55–51 | 2 |  |
| -50–46 | 1 |  |
| -45–41 | 6 |  |
| -40–36 | 7 |  |
| -35–31 | 11 |  |
| -30–26 | 2 |  |
| -25–21 | 8 |  |
| -20–16 | 2 |  |
| -15–11 | 15 |  |
| -10–6 | 20 |  |
| -5–0 | 20 |  |
| 0–5 | 15 |  |
| 6–10 | 11 |  |
| 11–15 | 7 |  |
| 16–20 | 2 |  |
| 21–25 | 1 |  |
| 26–30 | 9 |  |
| 31–35 | 61 |  |
| 36–40 | 54 |  |
| 41–45 | 22 |  |
| 46–50 | 15 |  |
| 51–55 | 9 |  |
| 56–60 | 10 |  |
| 61–65 | 6 |  |
| 66–70 | 4 |  |
| 71-75 | 5 |  |
| 76–80 | 3 |  |
| 81–85 | 2 |  |
